# Economic evaluations of screening and case-finding for Chronic Obstructive Pulmonary Disease (COPD): a systematic review

**DOI:** 10.1038/s41533-025-00467-1

**Published:** 2026-01-21

**Authors:** Yiwen Fan, Qiushi Chen, Hexi Sun, Till Bärnighausen, Chen Wang, Ting Yang, Simiao Chen

**Affiliations:** 1 Heidelberg Institute of Global Health, Faculty of Medicine and University Hospital, Heidelberg University, Heidelberg, Germany; 2 Harold and Inge Marcus Department of Industrial and Manufacturing Engineering, The Pennsylvania State University, University Park, PA USA; 3 School of Population Medicine and Public Health, Chinese Academy of Medical Sciences and Peking Union Medical College, Beijing, China; 4 Department of Pulmonary and Critical Care Medicine, Center of Respiratory Medicine, China-Japan Friendship Hospital, Beijing, China; 5 National Clinical Research Center for Respiratory Diseases, Beijing, China; 6 Chinese Academy of Engineering, Beijing, China

**Keywords:** Diseases, Health care, Medical research

## Abstract

Chronic obstructive pulmonary disease (COPD) imposes significant health and economic burdens globally. Screening and case-finding strategies are increasingly recognized as critical methods to enhance early diagnosis and management of COPD. It is important to understand the economic impact and cost-effectiveness of these strategies to inform the population health policies and real-world practice. In this study, we aim to summarize and compare the economic evaluations of COPD screening and case-finding strategies. We searched PubMed, EMBASE, Cochrane Library, and NHS economic databases for all published studies up to April 2025 that reported economic outcomes, including cost-effectiveness, budget impact, or cost analysis, related to screening and case-finding of COPD. Data extraction included study type, target population, methods, cost perspectives, and outcome measures. Findings were synthesized narratively. This systematic review was registered in PROSPERO (CRD42024516534). We identified 18 eligible studies that met the inclusion criteria, including 11 empirical and 7 modeling studies. A range of screening and case-finding approaches were evaluated, with most studies (n = 16) employing questionnaires either as standalone tools (n = 14) or for pre-screening purposes before the portable spirometer test (n = 8). Portable spirometers were also commonly used (n = 10). The economic outcome measures varied across studies, including cost per additional case detected, cost per quality-adjusted-life-year (QALY) gained, and program-level budget impact. Healthcare sector and payer’s perspectives were the most commonly adopted. While studies consistently suggested that targeted screening strategies were likely to be cost-effective, considerable heterogeneity in study designs, target populations, and economic measures limited direct comparisons between the strategies. COPD screening and case-finding showed potential of being cost-effective preventive strategies, particularly for high-risk groups. However, the lack of standardized descriptions for the details of the implemented strategies and the diverse outcome measures reported across existing studies limits the comparability between these strategies. Future research is needed to assess the long-term economic impact on healthcare systems and to explore personalized compared with one-size-fits-all screening strategies for COPD.

## Introduction

Chronic obstructive pulmonary disease (COPD) is a common lung condition characterized by chronic respiratory symptoms and irreversible damage to lung functions^1,2^. COPD is a rising cause of morbidity and mortality worldwide and is among the top four leading causes of death globally^3^, with 90% of these deaths occurring in low- and middle-income countries (LMICs)^4^. It also imposes a significant economic burden, with costs projected to reach $4.3 trillion globally from 2020 to 2050^5^. With the continued exposures to risk factors and an aging population, the global burden of COPD is expected to continue increasing in the coming decades^5,6^.

Despite being preventable and treatable, the awareness of COPD remains low^7–9^. Low diagnosis rates have been reported across countries—less than 3% patients in China^8^, 28% in the United States^10^, 33% in Canada^11^, and 14–41% in Spain^12^ were aware of their condition. To mitigate the substantial gap in disease awareness and diagnosis, screening and case-finding are the most commonly considered strategies to improve the diagnosis of COPD^13,14^. Haroon et al. reviewed case-finding approaches in primary care and found that all these strategies identified more undiagnosed COPD cases than usual care^15^. The two systematic reviews by the US Preventive Services Task Force (USPSTF) in 2016 and 2022 also confirmed that screening strategies using questionnaires or handheld spirometers could effectively identify previously undiagnosed COPD cases in primary care^16,17^. Timely diagnosis can enable earlier initiation of pharmacological and non-pharmacological interventions, which are essential to slow disease progression, reduce exacerbations, and improve quality of life^18–20^.

However, the cost of implementing such interventions at the population level may be nontrivial to the payers, which warrants careful economic evaluations to inform policymakers with such a substantial investment in population health. Most published studies, including the existing systematic reviews, related to COPD screening or case-finding have primarily focused on evaluating their effectiveness in a variety of clinical and community settings^15–17^. Few have systematically reported the economic impact of such intervention strategies and programs^21–24^. At the time of the analysis in this paper, no systematic review on the economic evaluation of COPD screening and case-finding was identified from the PROSPERO database. Therefore, given the promise of screening and case-finding for COPD with proven effectiveness in the literature, there is an imminent need to systematically review the existing evidence and data on the economic and cost-effectiveness outcomes of these interventions, which could provide valuable input for policymakers to consider their implementations in practice and to close the gap in the continuum of COPD care.

To fill this gap, in this study, we systematically reviewed published research on the cost and cost-effectiveness of COPD screening and case-finding strategies. Our goal is to review existing strategies, summarize current health economic evidence of these strategies and how they have been evaluated. Through this review, we aim to compare key differences in the screening and case-finding strategies across studies, examine how economic outcomes have been measured and reported, and identify gaps in current literature. These findings may help shape comprehensive reporting of cost-effectiveness results of COPD screening and case-finding strategies in future studies and inform policymakers with key considerations of choosing appropriate strategies across different healthcare settings.

## Methods

### Search strategy and selection criteria

The review was performed adhering to the PRISMA standards^25^ and the protocol for this review was registered on the PROSPERO prospective registry of systematic reviews (CRD42024516534).

We searched PubMed, EMBASE, Cochrane Library, and the National Health Service (NHS) Centre for Reviews and Dissemination (CRD) Database (including the Database of Abstracts of Reviews of Effects [DARE], the NHS Economic Evaluation Database [NHS EED], and the Health Technology Assessment [HTA] Database) through April 2025 with no restrictions on the start time. We conducted a systematic search using a combination of keywords and MeSH terms related to “chronic obstructive pulmonary disease (COPD)”, “screening or case-finding”, and “economic evaluation”. Terms within each concept category were combined using OR, and the categories were combined using AND. Searches were applied to the Title, Abstract, and Keywords fields where available. Detailed search terms used in the queries for each database are provided in Supplementary S1 Tables S1-S4. No language restrictions were applied.

We included all peer-reviewed studies that reported economic outcomes of COPD screening and case-finding strategies. Eligible approaches encompassed questionnaires, portable spirometers, risk prediction models, and broader disease management programs that aimed to facilitate early identification and diagnosis of COPD. We did not distinguish between screening and case-finding in our inclusion criteria, as both refer to strategies for detecting undiagnosed COPD. While some studies differentiate screening as targeting asymptomatic individuals and case-finding as targeting those with respiratory symptoms^23,26,27^, such a distinction is not consistent across studies, and there is no well-established definition of each with a clear distinction. Therefore, we included studies regardless of how they named the intervention, as long as the objective was for the early detection of COPD. While recent literature has suggested integrating screening for COPD with other diseases (e.g., lung cancer or cardiovascular disease) that share common risk factors may improve efficiency^28,29^, we restricted studies to the single-disease approach focusing on screening or case-finding for COPD only to maintain comparability of the COPD-related outcomes from the reviewed studies. We did not restrict the study to specific types of economic evaluation, including cost-effectiveness analysis, budget impact, or simple cost reports, and did not limit the research method to either empirical or modeling studies.

Two reviewers (YF and HS) independently screened the titles and abstracts of identified articles. Full-text papers were then obtained for articles deemed potentially eligible and assessed based on predefined inclusion and exclusion criteria. Any disagreements were resolved through discussion. Studies were included if they reported economic outcomes related to COPD screening or case-finding strategies. The following exclusion criteria were applied: (1) not a COPD screening or case-finding strategy; (2) not a strategy focused on a single disease; and (3) no cost-related information reported. Any disagreements were resolved through discussion. We also examined the full texts of potentially relevant articles and reviewed their reference lists to identify additional eligible studies.

### Data extraction and quality assessment

Data were extracted independently by each reviewer (YF and HS) on the study design, country, clinical setting, target population, screening and case-finding method, economic evaluation outcome measurement, study perspective, and currency year. If these data were not explicitly stated in the text, they were derived from the available information in the papers.

We assessed the methodological quality of all included economic evaluations using the Drummond 10-point checklist^30^, a widely used tool for appraising economic studies in health care^31–33^. This checklist evaluates whether key components of an economic evaluation are appropriately addressed. Its structured format facilitates consistent appraisal across studies and helps identify both strengths and limitations in study design and reporting. Each study was independently assessed by one reviewer (YF) against the ten checklist criteria. When there was uncertainty or ambiguity in scoring, a second reviewer (HS) was consulted, and consensus was reached through discussion.

The included studies were described and analyzed through a narrative systematic review. A synthesis table was created to summarize the findings based on the extracted data from reviewed studies. International currencies were converted to the US dollar values using the Purchasing Power Parity conversion factor from the World Bank^34^, and all cost estimates were adjusted to the 2025 value based on the Consumer Price Index in the US^35^ to facilitate comparisons across studies (Supplementary Table S5).

## Results

**Fig. 1:**
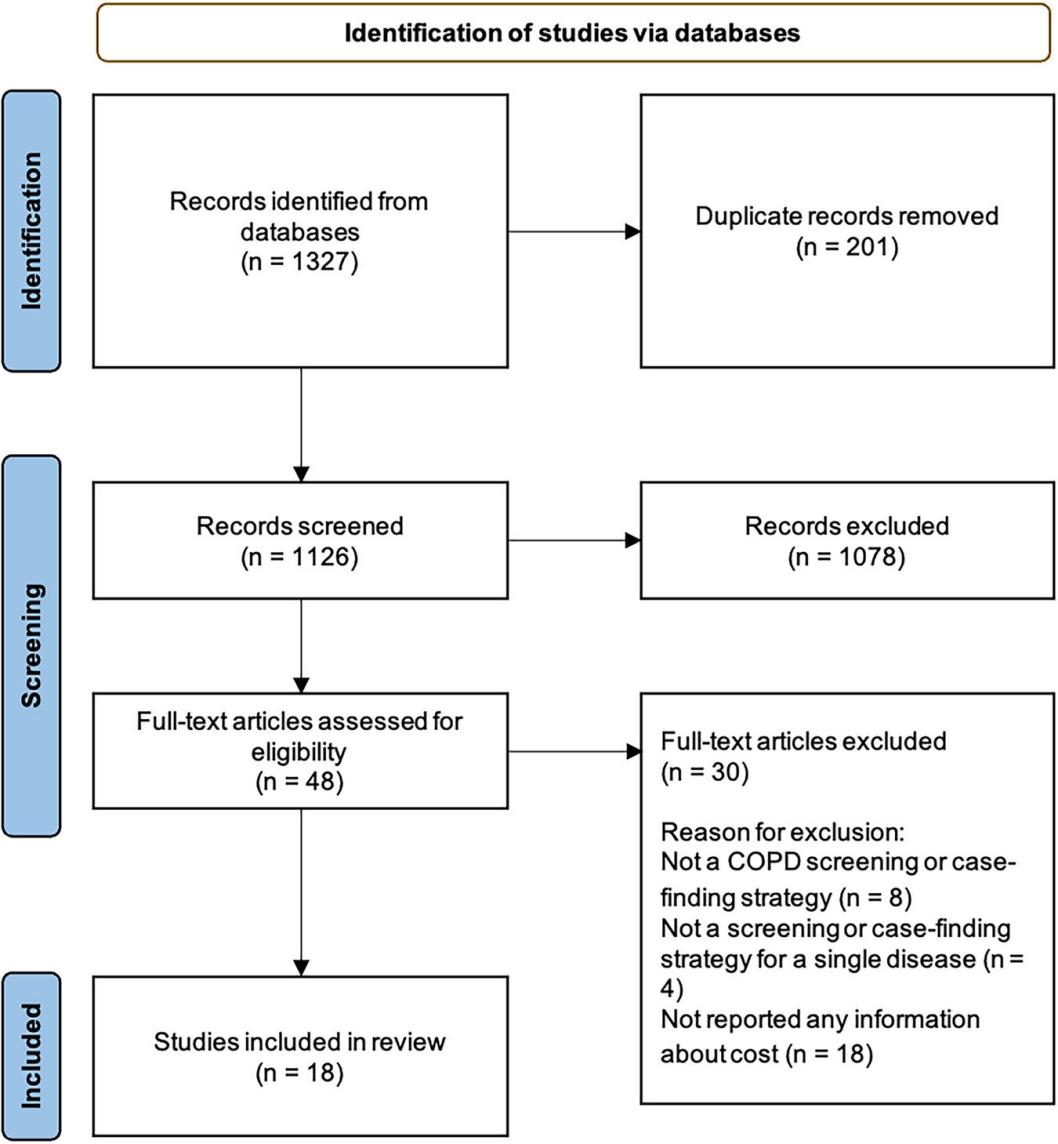
PRISMA flow diagram of the study selection. The figure shows the number of records included ane excluded at each stage of the search.

The search strategy identified 1,126 unique records and 18 studies met the inclusion criteria (Fig. 1), including eleven empirical studies and seven decision analytical modeling studies (Table 1). Specifically, empirical studies included two randomized controlled trials and nine observational studies; modeling studies included four Markov models, two discrete-event simulation models, and one microsimulation model. Most of the studies were conducted in high-income countries (n = 10), seven in LMICs, and one in multiple countries. While all studies focused on high-risk groups, the definition of high-risk groups varied by the risk factors considered across studies. The risk factors that have been frequently considered in the studies included smoking (n = 5), age (n = 14, typically 40 years and older), and the presence of respiratory symptoms (n = 2).

**Table 1:** Summary of empirical and modeling studies on economic evaluation of chronic obstructive pulmonary disease (COPD) screening and case-finding strategies.

Study	Study type	Study design and setting	Inclusion criteria or targeted population	Interventions for comparisons	Cost and cost-effectiveness outcome measures	Reported currency (year)	Main results (2025 US dollar values^a^)
Jones et al.^49^	Empirical	• Pilot implementation study • Mobile spirometry service in primary care practices (n = 98 patients, 7 practice sites) • Plymouth, UK	General practices in Plymouth known to own a spirometer	Structured COPD diagnostic and management service (staff education, spirometry clinics)	Incremental cost per case detected, cost outcome, and budget impact	UK pound (2004)	• Total cost of the program: £6,189 ($15,477) • Cost per new patient diagnosed: £107 ($268)
Konstantikaki et al.^26^	Empirical	• Cohort study • Comparison of open spirometry (n = 906) and case-finding (n = 201) programmes • Primary health care practices in semi-rural areas of Greece	Aged >30, residing near a primary health care practice	Open spirometry programme based on public invitation compared with a case-finding programme where spirometry was offered to subjects selected by properly trained primary care physicians using a screening questionnaire	Incremental cost per case detected	Euro (2008)	• Cost per diagnosis: €134 ($284) for open spirometry and €78 ($165) for case-finding • Cost per new diagnosis: €173 ($367) for open spirometry and €102 ($216) for case-finding
Thorn et al.^64^	Empirical	• Cross-sectional study • Lung function pre-screening (n = 305) • Urban and rural primary health care centres in Sweden	Aged 45–85 years, smoking history>15 pack-years	Mini-spirometer pre-screening compared with standard spirometry without pre-screening	Incremental cost per case detected	Swedish Kronor (2009)	• Cost per additional case detected for no pre-screening compared with COPD-6 pre-screening: SEK 2559 ($430) at a COPD-6 FEV1/FEV6 cut-off of 78%
Dirven et al.^51^	Empirical	• Observational study with mixed methods • Population-based approach for the early detection (n = 831) • General practices in Netherland	Aged 40-70 years without prior COPD diagnosis	Respiratory Health Screening Questionnaire (RHSQ)	Cost outcome and budget impact	Euro (2013)	• Cost per practice for three months: €460 ($795)
Haroon et al.^21^	Empirical	• Pilot RCT • Targeted case-finding (n = 815) versus opportunistic case-finding (n = 819) • General practices in the West Midlands, UK	Ever-smokers aged 35–79 years with no history of COPD or asthma	Targeted case-finding with questionnaires posted to patients compared with opportunistic case-finding with questionnaires provided at routine general practice appointments	Incremental cost per case detected	UK pound (2011)	• Cost per case detected: £425 ($867) in the targeted case-finding and £242 ($494) in the opportunistic case-finding
Jithoo et al.^47^	Empirical	• Cross-sectional study • Different case identification algorithms from the Burden of Obstructive Lung Disease (BOLD) study (n = 9,390) • 14 countries	Aged ≥40 years	Various strategies using a questionnaire and/or peak expiratory flow (PEF)	Incremental cost per case detected	Not applicable	• Cost per case detected: 27.9 resource units using PEF only (the most cost-effective screening strategy), where resource unit=10 min of nurse/technician time
Tawara et al.^43^	Empirical	• Prospective cohort study • Systematic COPD intervention program (n = 8,878) • Communities in Matsuura, Japan	Aged 50-89 years inhabitants	Systematic intervention using a self-administered eleven-item pre-interview questionnaire (11-Q) compared with no intervention	Cost outcome and budget impact	Japanese yen (2013)	• Initial screening cost: 2,240,000 yen ($30,514) • Annual action costs: approximately 400,000 yen ($5,449) • Significantly lower COPD medical costs per patient in the intervention region compared to the region with no intervention (49.3 ± 25.2 yen/month vs 82.7 ± 37.0 yen/month)
Jordan et al.^22^	Empirical	• Clustered RCT • Targeted case finding (n = 32,789) versus routine practice (n = 42,029) • General practices in the West Midlands, UK	Aged 40-79, ever-smokers, without a previously recorded diagnosis of COPD	Opportunistic case-finding with screening questionnaire provided during general practice consultation and active case-finding with screening questionnaire also provided by mail compared with routine care	Incremental cost per case detected	UK pound (2013)	• Cost per additional case detected compared with routine practice: £333 ($661) in active case-finding and £376 ($746) in opportunistic case-finding
Pan et al.^45^	Empirical	• Cross-sectional study • COPD screening tests and combinations (n = 2445) • Urban and rural community health centres in four municipalities of China	Aged ≥40 years, community residents who attended community health centres	Four questionnaires, peak flow, microspirometry, and their combination (22 strategies in total)	Incremental cost per case detected	UK pound (2019)	• Cost per additional true case detected compared with no screening: £18 ($34) for microspirometry, £19 ($36) for Peak flow, and £29 ($55) for combination of Chinese Symptom-Based Questionnaire (C-SBQ) and microspirometry
Martins et al.^46^	Empirical	• Cross-sectional study • COPD screening tests and combinations (n = 1162) • Basic health units in São Bernardo do Campo, Brazil	Aged ≥40 years registered hypertensive patients who attended routine consultations	Four questionnaires, peak flow, microspirometry, and their combination (14 strategies in total)	Incremental cost per case detected	UK pound (2019)	• Cost per additional true case detected compared with no screening: £63-80 ($124-151)
Mohan et al.^52^	Empirical	• Cross-sectional study • COPD screening tools (n = 10,709) • Nepal, Peru, and Uganda	Aged ≥40 years residents	Three different screening strategies including CAPTURE combined with peak expiratory, COLA-6 combined with peak expiratory, and LFQ	Incremental cost per case detected	US dollar (2019)	• Cost per correct positive diagnosis: $44-56 ($55-71) in Nepal, $107-123 ($135-155) in Uganda, and $1,483-1,886 ($1,869-2,376) in Peru
Lambe et al.^23^	Modeling	• Markov model • Model cohort size: not applicable^b^ • Time horizon: 50 years • Primary care setting in the UK	Aged ≥50 years, ever smokers without prior COPD diagnosis	Respiratory screening questionnaire compared with routine practice	Incremental cost per QALY	UK pound (2015)	• ICER: £16,596 ($32,599)/QALY • High probability of cost-effectiveness at the £20,000 ($39,286)/QALY threshold
Du et al.^44^	Modeling	• Decision tree and Markov model • Model cohort size: not applicable^b^ • Time horizon: 15 years • County health centers in China	Mainly smokers aged over 45 and with long-term exposure to secondhand smoke, underlying lung diseases, a family history of lung diseases, or respiratory symptoms	COPD-population screener (COPD-PS) questionnaire compared with no screening	Incremental cost per QALY	Chinese yuan (2019)	• ICER: ¥6,366 ($1,976)/QALY, below a ¥70,889 ($22,002)/QALY threshold
Johnson et al.^48^	Modeling	• Discrete-event simulation model • Model cohort size: 1000 • Time horizon: 20 years • Primary care setting in Canada	Aged ≥40 years general population	Sixteen case detection strategies that combined eligibility (based on age, smoking history, or symptoms), methods (CDQ, screening spirometer), and time intervals (every 3 or 5 years) compared with no case detection	Incremental cost per QALY	Canadian dollar (2019)	• ICER: $16,251-46,956 ($16,813-48,581)/QALY for all scenarios compared with no detection, below $50,000 ($51,730)/QALY threshold • ICER: $19,632 ($20,311)/QALY for all patients aged ≥ 40 years who received the CDQ at 3-year intervals, which was the most effective scenario
Qu et al.^27^	Modeling	• Decision tree and Markov model • Model cohort size: 1000 • Time horizon: life-time • Primary care setting in China	Chronic bronchitis (CB) patients	Portable spirometer or questionnaire compared with no screening	Incremental cost per QALY	Chinese yuan (2018)	• ICER: < 0 (cost-saving; positive QALY, negative cost) for both questionnaire and portable spirometer screening compared with no screening
Mountain et al.^50^	Modeling	• Discrete-event simulation model • Model cohort size: 19.8 million • Time horizon: Year 2022 to 2026 • Primary care setting in Canada	Aged ≥40 years general population	Eight primary care–based case detection strategies combined eligibility (based on age, smoking history, or symptoms) and methods (CDQ or screening spirometer) compared with no case detection	Cost outcome and budget impact	Canadian dollar (2021)	• Budget impact: increase $128-423 ($133-440) billion for different case detection strategies over a 5-year period
Chen et al.^24^	Modeling	• Microsimulation model • Model cohort size: 1,000,000 • Time horizon: life-time • Community setting in China	Aged ≥35 years general population	Twelve screening policies differed by frequency and method (COPD-SQ alone or with portable spirometer) compared with no screening	Incremental cost per QALY	US dollar (2022)	• ICER: $8,034-13,209 ($9078-14,926)/QALY for scenarios compared with no screening, below $38,441 ($43,438)/QALY threshold (3-time GDP per capita) • ICER: $13,671 ($15,448)/QALY for annual two-step screening, which was the most cost-effective scenario
Zhang et al.^61^	Modeling	• Markov model • Model cohort size: 100,000 • Time horizon: life-time • National program in community setting of China	Aged ≥40 years general population	COPD-SQ followed by portable spirometer compared with no screening	Incremental cost per QALY	US dollar (2023)	• ICER: $5,679 ($6,020)/QALY, below $11,814 ($12,523)/QALY threshold (1-time GDP per capita)

*COPD*, chronic obstructive pulmonary disease; *RCT*, randomized controlled trial; *QALYs*, quality-adjusted life years; *CAPTURE*, COPD assessment in primary care to identify undiagnosed respiratory disease and exacerbation risk; *COLA-6*, COPD in low- and middle-income countries assessment-6; *LFQ*, lung function questionnaire; *CDQ*, COPD diagnostic questionnaire; *COPD-SQ*, COPD screening questionnaire; *ICER*, incremental cost-effectiveness ratio.

^a^Values in parentheses were converted to US dollars of the corresponding year^34^ and adjusted to 2025 US dollars using the US consumer price index^35^.

^b^Markov models usually focus on calculating the probability distribution of health states over time, and thus do not require a specified cohort size.

### Quality assessment

**Fig. 2:**
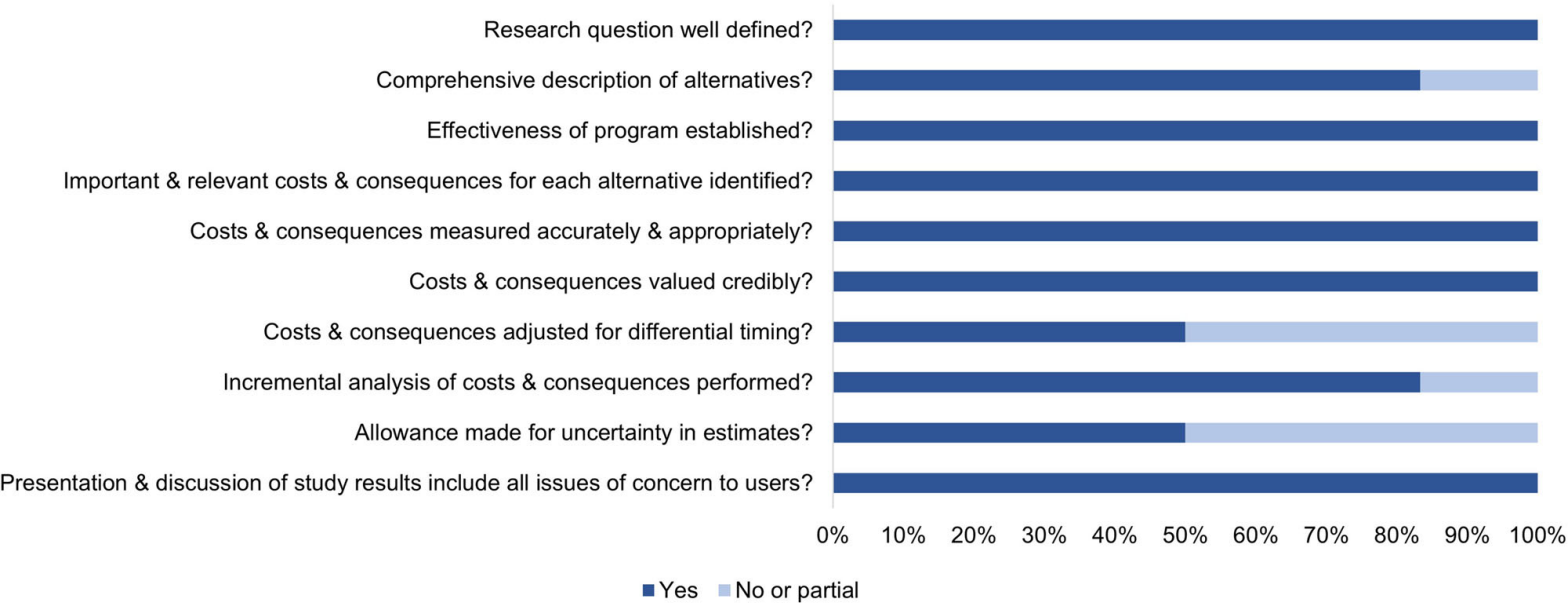
Quality assessment of included studies with the Drummond 10-point checklist. This figure depicts the proportion of included studies (n=18) that met each criterion of the Drummond 10-point checklist.

All studies clearly defined their research questions, established effectiveness, identified relevant costs and consequences, and provided a comprehensive presentation and discussion of results, according to the Drummond 10-point checklist (Fig. 2). Among eleven empirical studies, only three studies (27%) addressed adjustment for differential timing, which may be because most studies were one-time assessments conducted for the screening and case-finding program over a short time period; only two studies (18%) addressed uncertainty in estimates, and most empirical studies only reported point estimates of total health and economic outcomes without formal statistical analyses to quantify their uncertainty. All seven modeling studies conducted at least one form of one-way, scenario, or probabilistic sensitivity analyses, thereby addressing uncertainty in parameter estimates to varying degrees. Overall, eight studies (44.4%) fully met all ten checklist criteria (see Supplementary Table S6).

### Tools for screening and case-finding

The reviewed studies employed a variety of tools for COPD screening and case-finding, including portable spirometers and various questionnaires. In all except two studies (n = 16), questionnaire-based approaches were employed as standalone screening tools (n = 14), and/or as pre-screening methods (n = 8) to identify individuals at high risk of COPD who would then undergo the next step of screening (Supplementary Table S7). The most commonly used questionnaires among the included studies were the COPD Screening Questionnaire (COPD-SQ) (n = 4) and the COPD Diagnostic Questionnaire (CDQ) (n = 5). The COPD-SQ was developed and validated in China and has demonstrated adequate accuracy for screening in the Chinese population^36^; the CDQ was originally developed in the UK and the US and has since been frequently applied and externally validated in various international settings^37,38^. Other screening questionnaires used in the included studies and also in practice include the Lung Function Questionnaire (LFQ)^39^, COPD Population Screener (COPD-PS)^40^, and COPD Assessment in Primary Care To Identify Undiagnosed Respiratory Disease and Exacerbation Risk (CAPTURE)^41,42^. Although they differ in the validation populations and reported sensitivity and specificity, these screening questionnaires share a common structure, typically including information on age, smoking history, and respiratory symptoms such as cough, dyspnea, and wheeze (see detailed comparisons in Table 2). Another widely used tool for screening and case-finding was the portable spirometer (n = 10), which was adopted as a standalone test (n = 6) or combined with a questionnaire (n = 8). All included studies confirmed COPD diagnosis using confirmatory spirometry, except for two studies^43,44^ that reported conducting more comprehensive pulmonary function testing, which served as the case definition method for identifying true positive cases.

**Table 2:** Summary of COPD screening questionnaires used in the included studies.

Questionnaire name	Abbre-viation	Year of publication	Initial validation setting	Key items	Number of items included	Reported sensitivity	Reported specificity	Used in the studies included in the review
COPD Diagnostic Questionnaire^84^	CDQ	2006	Primary care setting in UK and US	Age, BMI, smoking history, respiratory symptoms (cough, sputum, dyspnea, wheeze), allergies, activity limitation	9	54-82%	58-88%	Pan et al.^45^ Dirven et al.^51^ Martins et al.^46^ Johnson et al.^48^ Mountain et al.^50^
Symptom-Based Questionnaire^37^	SBQ	2006	Primary care setting in UK and US	Age, BMI, smoking history, respiratory symptoms (cough, phlegm, wheeze), allergies	8	80.40%	72.00%	Martins et al.^46^
COPD Eleven-Item Pre-Interview Questionnaire^85^	11-Q	2006	Primary care setting in Japan	Age, smoking history, respiratory symptoms (phlegm, cough, dyspnea, wheeze), family history, childhood history, underweight/overweight, exposure	11	66.70%	76.80%	Tawara et al.^43^
COPD Population Screener Questionnaire^40^	COPD-PS	2009	Primary care setting in US	Age, smoking history, respiratory symptoms (cough, breathlessness), activity limitation	5	84.40%	60.70%	Konstantikaki et al.^26^ Du et al.^44^
Lung Function Questionnaire^86^	LFQ	2009	US NHANES III	Age, smoking history, respiratory symptoms (phlegm, dyspnea, wheeze)	5	73.20%	58.20%	Mohan et al.^52^
COPD Screening Questionnaire^36^	COPD-SQ	2013	Community setting in China	Age, BMI, smoking pack-years, respiratory symptoms (cough, dyspnoea), family history, exposure to biomass smoke	7	60.60%	85.20%	Pan et al.^45^ Martins et al.^46^ Chen et al.^24^ Zhang et al.^61^
COPD Assessment in Primary Care To Identify Undiagnosed Respiratory Disease and Exacerbation Risk^87^	CAPTURE	2016	Primary care setting in US	Exposure, breathing problems, tiring, respiratory illnesses, activity limitation	5	95.70%	44.40%	Pan et al.^45^ Martins et al.^46^ Mohan et al.^52^
Chinese Symptom-Based Questionnaire^88^	C-SBQ	2016	Hospital setting in China	Age, BMI, smoking, (cough, wheeze, dyspnea), allergies, exposure, childhood history	11	82.45%	72.87%	Pan et al.^45^
COPD in Low- and Middle-Income Countries Assessment^89^	COLA-6	2020	Community setting in Uganda	Age, smoking history, exposure, respiratory symptoms (phlegm, wheeze), activity limitation, hospitalization	7	43.00%	96.00%	Mohan et al.^52^

The Respiratory Health Screening Questionnaire (RHSQ) used in Dirven et al.^51^ is identical to the COPD Diagnostic Questionnaire (CDQ) and thus not listed separately.

*COPD*, chronic obstructive pulmonary disease; *BMI*, body mass index; *NHANES*, the national health and nutrition examination survey.

Several studies combined these tools to potentially enhance the accuracy and accessibility of screening strategies. For instance, Pan et al.^45^ and Martins et al.^46^ evaluated the accuracy and cost-effectiveness of four different COPD screening questionnaires, two portable spirometers, and all their combinations (i.e., resulting in a total of 4*2 = 8 combined strategies). These studies found that combinations increased the screening costs compared to single-tool approaches but improved specificity and reduced false-positive referrals to further diagnostic tests. They highlighted that in LMICs, where diagnostic spirometry is often unavailable or unreliable in primary care, accurate pre-referral combined screening could help improve access and optimize resource use. Findings from a modeling study by Chen et al.^24^ further supported this point from a broader cost perspective. Their analysis showed that combined screening approaches resulted in marginally lower health gains than using a questionnaire alone, but they led to substantially lower total program costs, including downstream diagnostic and management expenses. The cost savings were primarily attributed to the higher specificity of the combined strategy, which reduced unnecessary diagnostic procedures. On the other hand, Jithoo et al.^47^ argued that combined screening that targets high-risk populations is less efficient than universally applying peak expiratory flow (PEF), because it could be measured regularly through simple portable devices. While acknowledging the clinical value of identifying high-risk individuals, they concluded that such approaches may lead to higher costs and fewer cases detected.

### Screening and case-finding strategies

The studies varied widely in their implementations of screening and case-finding strategies. In terms of screening and case-finding frequency, the empirical studies typically evaluated one-time screening and case-finding interventions, focusing on the immediate costs and outcomes associated with detecting new cases of COPD. In contrast, a part of the modeling studies (n = 3) assessed the long-term cost-effectiveness of repeated screening and case-finding strategies with a frequency ranging from every 10 years to annually and over extended periods spanning from 5 years to life-time based on their cumulative impact on healthcare costs and health outcomes^23,24,48^.

In terms of the comprehensiveness of the implemented strategies, several studies considered comprehensive disease management programs with extended follow-up, beyond focusing on the screening and detection step only. Jones et al.^49^ implemented a three-month structured COPD diagnostic and management service that included staff training on the use of spirometry, data interpretation, and COPD management. Konstantikaki et al.^26^ compared an open spirometry program based on public invitation with a targeted case-finding approach, where primary care physicians selected patients for spirometry based on specific risk factors. While Tawara et al.^43^ implemented a more systematic intervention in Japan that included ongoing detection, examination, education, and treatment interventions, performed follow-up examinations or respiratory lessons yearly, and supported the health maintenance of each patient.

### Economic outcome measures

Ten studies reported health economic outcomes from either a healthcare sector perspective (n = 5) or a payer perspective (n = 5), while eight studies (n = 8) did not report their perspective of analysis explicitly. We summarized the primary outcome measures reported from these studies into the following three main categories: cost outcome, incremental cost per additional case detected, and incremental cost per quality-adjusted life year (QALY) gained.

#### Cost outcome and budget impact

Studies varied substantially in the cost components that were considered. All studies accounted for screening delivery costs, including expenses related to screening tools and labor costs associated with staff time. Eight studies (n = 8) included program setup costs, such as personnel training and administrative coordination. Seven studies (n = 7) incorporated post-screening and disease management costs, covering treatment, hospitalization, and long-term maintenance. However, none of the studies explicitly considered patient-incurred time costs, productivity losses, or broader societal impacts beyond the healthcare sector. A summary of the cost components included in each study is provided in Supplementary Table S7.

Four studies reported total program costs or conducted formal budget impact analyses as their primary outcome. For example, Mountain et al.^50^ conducted a budget impact analysis using a modeling approach in Canada, incorporating a 5-year projection of a primary care-based case detection program from the payer’s perspective. In addition to case detection costs, their analysis included administrative expenses and patient-level costs. In contrast, studies like Jones et al.^49^ in the UK and Dirven et al.^51^ in the Netherlands focused on short-term implementation costs from a health care sector perspective. These studies reported total costs over a 3-month period, covering training, staff time, and equipment use, without incorporating downstream patient care costs.

#### Cost-effectiveness measured by the incremental cost per case detected

**Fig. 3:**
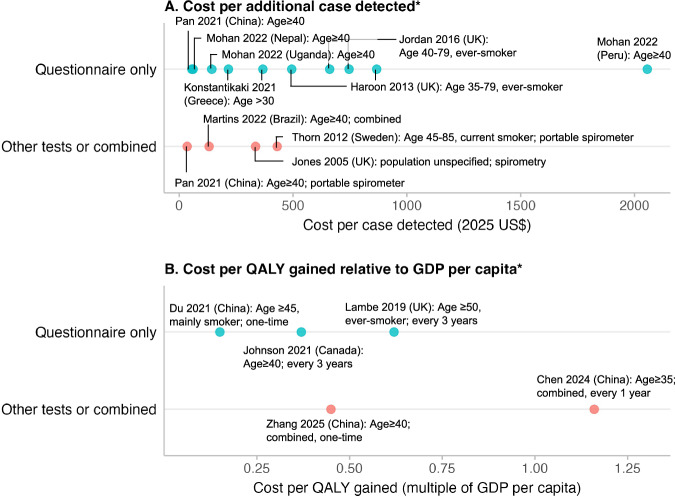
Summary of cost-effectiveness results of COPD screening and case-finding strategies from included studies. Panels show (**A**) the cost per case detected and (**B**) the cost per QALY gained relative to the country’s GDP per capita. All costs were standardized to US dollars in 2025 value. When multiple strategies using the same method but different tools were evaluated, only the most cost-effective strategy is displayed. *Incremental costs are compared with usual care or no screening, except for Chen et al.^24^, which compared with the preceding non-dominant scenario.

Nine studies evaluated the number of correct cases detected to measure the effectiveness of screening of various strategies in different clinical settings and reported the incremental cost per additional case detected (Fig. 3). For instance, Martins et al.^46^ reported an incremental cost-effectiveness ratio (ICER) ranging 66–81 UK£ (equivalent to $107–131 in 2025 USD value) per additional true case detected between non-dominated strategies among different combinations of questionnaires and portable spirometers in Brazil. Whereas Pan et al.^45^ expanded upon these screening strategy combinations by including additional questionnaire and portable spirometer combinations (with 22 combinations in total), resulting in ICERs in a range of 18–29 UK£ (equivalent to $29–47 in 2025 USD value) per additional true case detected in the Chinese clinical setting. Mohan et al.^52^ examined the cost per correct positive diagnosis from screening strategies compared with no screening in three countries: approximately $50 in Nepal, $120 in Uganda, and over $1,500 in Peru, (equivalent to $61, $143, and $2,056 in 2025 USD value, respectively) which is the highest among all included studies.

#### Cost-effectiveness measured by the incremental cost per QALY

Six studies used modeling techniques to estimate the cost per QALY gained, and all of them concluded that COPD screening and case-finding strategies are cost-effective compared with no screening or routine care. Most studies incorporated direct medical costs, including screening procedures, treatment, and disease management. However, only a few^24,48^ considered program implementation setup costs such as training or administrative infrastructure. Health utilities used to estimate QALYs were generally stratified by COPD severity and adjusted for exacerbation or treatment. Utility values were primarily derived from published literature^53–56^ and national cohort data, such as the Birmingham cohort in the UK^57^ and the National Enjoying Breathing Program in China^58^. Details on the health utility values are summarized in Supplementary Table S8.

Different willingness-to-pay (WTP) thresholds based on local or international guidelines were applied to determine the cost-effectiveness. For instance, Johnson et al.^48^ used the Canadian threshold of $50,000 per QALY^59^; Lambe et al.^23^ adopted the UK NICE threshold of £20,000–£30,000^60^; and four studies (Du et al.^44^, Qu et al.^27^, Chen et al.^24^, and Zhang et al.^61^) applied one- to three-times GDP per capita as the WTP, following the WHO recommendations^62^. In addition to variation in threshold selection, the included modeling studies also differed in their modeling approaches. Included studies employed either a Markov model (n = 4) or individual-level microsimulations (n = 2). For example, Lambe et al.^23^ used a Markov decision-analytic model with a 50-year horizon to evaluate respiratory-questionnaire screening adopted every three years. They found that offering questionnaires during routine practice visits to eligible patients yielded an ICER of £16,596 per QALY compared with routine practice, which was below the threshold for determining cost-effectiveness, ranging between £20,000-30,000 WTP threshold. Meanwhile, Chen et al.^24^ used a microsimulation with a lifetime horizon to compare twelve population-based screening policies (questionnaire alone or plus portable spirometer, one-time or repeated every 1, 2, 5, or 10 years) in the general population aged ≥ 35 years in China. All policies were deemed to be cost-effective under a three-time GDP per capita threshold compared with no screening. Although the choice of WTP threshold influences comparability, the consistent finding across studies, that ICERs remained below locally accepted thresholds, provides robust support for the long-term cost-effectiveness of COPD screening and case-finding.

## Discussion

Previous guidelines have advised against routine COPD screening for asymptomatic adults in the general primary care population, largely due to concerns over the cost and efficiency of spirometry-based screening^17,63^. However, these recommendations may have overlooked the benefits of alternative easy-to-implement screening instruments such as questionnaires and portable spirometers. With the growing burden of COPD globally, the economic value of screening and case-finding is gaining renewed attention, particularly in the context of large-scale implementation. To synthesize the existing evidence from the literature on this critical issue, we conducted a systematic review and included 18 studies that evaluated the cost impact and the cost-effectiveness of various COPD screening and case-finding strategies. Most studies showed that these strategies could offer cost-effective solutions, contributing to both improved case identification and more sustainable management of COPD at the population level.

Our review revealed substantial variations in the estimated cost per additional case detected across empirical studies. Higher costs per case were generally reported in studies from high-income countries such as the UK and Sweden^21,22,64^, whereas lower costs were observed in LMICs, including China^45^, Nepal, and Uganda^52^. Notably, the estimate from Peru was markedly higher than in any other country, which may be due to the lower COPD prevalence among the screened population (reported as 2.7% compared with 18.2% in Nepal and 7.4% in Uganda^52^) and the much lower population density, less than one-tenth of that in the Nepal site^65^, resulting in possibly greater personnel or logistical challenges associated with program implementation. Differences in risk factor profiles did not appear to systematically explain the variation in reported costs, likely due to substantial heterogeneity in settings, study designs, and cost estimation methods across studies. Nonetheless, screening among higher-risk populations, such as smokers or individuals with respiratory symptoms, is expected to be more cost-efficient than screening in the general population or among those without such risk factors, as it increases the yield of true positives without proportionally raising program costs. Given the limited number of studies, we did not observe a clear advantage of questionnaires or portable spirometer-based approach in terms of their cost per case detected. While portable spirometers generally incur higher costs, they also usually offer greater screening accuracy^66^, implying a trade-off between cost and performance of the test. It is also important to note that, unlike the cost-effectiveness measure of cost per QALY gained, there is no established threshold or range for cost per case detected. As a result, cost-effectiveness results should be interpreted and discussed based on relative comparisons of this ratio across studies reporting the same outcome, while taking into account the differences in the screening strategies and settings of these studies.

Despite differences in modeling structures, target populations, and outcome definitions, modeling studies consistently found COPD screening and case-finding to be cost-effective when assessed against appropriate WTP thresholds. These studies suggest that early detection combined with timely management can reduce the long-term costs associated with advanced disease, including frequent exacerbations and hospitalizations. While the above findings support the economic value of COPD screening, experts pointed out that large-scale implementation in primary care may still be constrained by workforce shortages and the heavy workload of physicians^67^. In real-world settings, involving other healthcare professionals, such as pharmacists, nurses, and community healthcare workers, in implementing screening and case-finding policies has been proposed as a practical approach to enhance its feasibility and scalability.

We also identified a significant variability in the implementation and the screening tools used in the COPD screening and case-finding strategies. Targeted screening programs conducted in primary care focused on high-risk groups could offer an economically viable approach to improving COPD diagnosis rates^21,22^. Several studies identified the use of pre-screening questionnaires followed by a portable spirometer as a particularly effective strategy to reduce unnecessary diagnostic tests and enhance the cost-effectiveness^45,46,48^. Additionally, integrated care interventions that combine case-finding with long-term management have shown potential to improve health outcomes and control costs by enabling early detection and continuous management, thereby preventing disease progression and costly exacerbations^43,49^. Given the diversity of study designs and implementation models, policymakers should carefully consider their target populations and healthcare systems, and compare the relative benefits and feasibility of different strategies.

Across the included studies, detailed breakdowns of cost components or cost drivers were not consistently and systematically reported, limiting direct comparison of cost structures. However, several recurrent factors appeared to substantially influence both costs and outcomes. Staff time was frequently identified as a major cost driver, not only for conducting screening procedures but also for preparatory tasks such as participant recruitment, invitation, and post-screening data management^22,51^. Sensitivity analyses from the included studies further indicated that screening uptake and risk profiles of the target population were key determinants of cost-effectiveness^22–24^. Higher participation rates and focusing on populations with more stringent risk factors, such as older age or tobacco exposure, tend to improve cost-effectiveness. From an implementation perspective, policymakers should estimate workforce requirements based on the expected target population size and consider measures to enhance screening uptake (e.g., community outreach, posters, or repeated invitations). While targeting high-risk groups may improve efficiency, it could also risk missing undiagnosed cases among lower-risk individuals, which requires careful program design and evaluation by implementers.

All included studies adopted a healthcare sector or payer perspective, and none took a broader societal perspective that captures non-medical impacts such as patient time and productivity losses. Evidence from COPD burden studies indicates that indirect societal costs can be substantial^5^, accounting for approximately 27–61% of total COPD-related costs^68^. Typical COPD symptoms, such as fatigue, dyspnea, cough, and wheeze, can reduce workforce participation and increase absenteeism and disability, while family caregivers may also experience work loss and reduced productivity^69^. Inclusion of these indirect cost components could influence the cost-effectiveness of COPD screening in both directions: early detection could prevent exacerbations and hospitalizations, and thus reduce long-term productivity losses and caregiver burden, thereby improving the cost-effectiveness; on the other hand, false positives from screening tests could result in unnecessary follow-up visits and thus additional opportunity cost of patients’ time, thereby decreasing the cost-effectiveness. In line with the recommendation by the Second Panel on Cost-Effectiveness in Health and Medicine^70^ that all cost-effectiveness analyses report both a healthcare sector and a societal reference case, future studies evaluating the cost-effectiveness of COPD screening strategies should therefore consider incorporating societal perspectives to enhance comparability and provide a more comprehensive understanding of the economic value of COPD screening and case-finding.

We observed that many studies did not clearly report the participant recruitment methods and the contextual settings in which screening and diagnostic tests were implemented. However, these elements are essential to understanding the design and implementation of screening strategies, as they often vary by country and healthcare system structure and may decisively shape the feasibility of implementation. For example, studies from the UK^22,23^, Sweden^64^, and Canada^48^ typically relied on routine primary care visits for recruitment and testing, with screening and follow-up diagnostic tests also conducted within the same visit. While in countries like China, where regular healthcare visits were less common, recruitment often required more proactive community outreach^24^. Furthermore, community-level health facilities often lacked the capacity to perform diagnostic spirometry, requiring referrals to higher-level hospitals, which means screening and diagnostic testing might not occur during the same visit^61^. This could introduce logistical challenges, limit follow-up compliance, and increase the overall cost of delivering screening and diagnostic services^71^. Despite these meaningful differences, studies rarely described or distinguished these contextual features in sufficient detail.

This variation reflects a broader conceptual ambiguity in how COPD screening and case-finding are defined and differentiated, which contributes to the ongoing debate surrounding its value. Critics of screening often defined it as confirmatory spirometry applied to all eligible individuals, regardless of symptoms^72^. In contrast, proponents argued that the definition of screening should include identifying high-risk populations through questionnaires or portable spirometers, followed by confirmatory spirometry in those who screened positive^18^. Consistent with this ambiguity, our review found that included studies often used the terms ‘screening’, ‘case-finding’, and ‘case detection’ inconsistently, with few providing comprehensive descriptions of their study designs, reinforcing the existing confusion. To improve clarity, we suggest standardizing the reporting of study design elements in future research, by explicitly stating key attributes such as study type, risk factors, recruitment method, screening tools and setting, screening threshold, diagnostic test and setting, and frequency (see detailed in Table 3). We also applied this structure to summarize the included studies (see Supplementary Table S9).

**Table 3:** Suggested reporting structure for economic evaluation studies on chronic obstructive pulmonary disease (COPD) screening and case-finding strategies.

Study Design Element	Definition and Examples
Study type	Type of economic evaluation conducted, such as empirical (e.g., trial-based or observational) or decision-analytic modeling (e.g., Markov models, microsimulations)
Risk factors considered	Characteristics used to define the high-risk population, such as age, smoking status, respiratory symptoms, or comorbidities like hypertension
Recruitment method	How the target population was identified or invited to participate, e.g., during routine clinic visits, via advertisements, or through active outreach in communities
Screening tool and setting	Specific tools used for initial screening (e.g., questionnaire, portable spirometer) and their application setting (e.g., mailed, online, primary care, or community clinics)
Positive threshold for screening	Criteria used to define a positive screening result that qualifies an individual for further diagnostic testing
Diagnostic test and setting	Type of confirmatory test used (typically spirometry) and the healthcare setting in which it was performed (e.g., primary care, hospital outpatient)
Frequency	Whether the screening strategy was conducted as a one-time activity or repeated periodically (e.g., annually, every 3 years)

Most included studies evaluated broad screening and case-finding strategies, but few explicitly addressed the potential of personalized approaches. While one-size-fits-all designs are easier to implement, they may be suboptimal in settings where COPD risk factors are unevenly distributed or healthcare resources are limited. With growing access to real-world health data and advanced analytical methods, precision public health (PPH) offers new opportunities to tailor screening based on individual risk profiles^73^. For example, machine learning (ML) has been successfully applied to the early detection and diagnosis of diseases such as cancers^74,75^, hypertension^76^, and diabetes^77^, and studies have also demonstrated the clinical potential of ML-based COPD screening questionnaires^78,79^. A recent review further highlighted that ML models can integrate diverse data sources to improve early COPD detection and reduce unnecessary testing^80^. While conceptually promising, these strategies have yet to be systematically integrated into economic evaluation frameworks, leaving important opportunities for future research. Beyond the disease-specific approach, future research could also explore the potential of multimorbidity-oriented, integrated screening strategies. Recent studies have suggested that integrating COPD screening with lung cancer, cardiovascular^28,29^, or asthma^81^ programs could improve diagnostic yield and efficient resource use by leveraging shared risk factors such as smoking or respiratory symptoms. However, the economic evidence for such integrative models remains limited. Evaluating their feasibility and cost-effectiveness will be essential to inform the next generation of COPD and multimorbidity management.

Within the 18 studies included in this review, five are based in China, four in the UK, two in Canada, and no studies are from the US. In this context, it is worth noting that the USPSTF’s recommendation against screening for COPD in asymptomatic adults, as they concluded that such screening provides no demonstrated net benefit in reducing morbidity or mortality, while potentially increasing opportunity costs within primary care^16,17^. However, they also acknowledge the importance of active case-finding among high-risk individuals such as current smokers, who have a substantially higher prevalence and incidence of COPD than non-smokers^82,83^. This highlights opportunities for more accessible, risk-based, and cost-efficient screening approaches. In this context, easy-to-implement instruments such as questionnaires and portable spirometers may provide cost-efficient alternatives for population-level implementation.

This review has several limitations. First, the quality assessment of the included economic evaluations was conducted using only the Drummond 10-point checklist. While this tool is widely used for assessing the quality appraisal of economic evaluations and is easy to interpret, it provides limited insight into the technical quality of decision-analytic models, such as model structure, data sources, sensitivity analysis, or validation. There exist checklists for assessing technical qualities for modeling studies, but they may not be applicable to the empirical studies included in our review. Therefore, we followed the Drummond checklist as a general guide and supplemented it with more details on the key components of study design to assess the quality of the included studies. Second, there existed substantial heterogeneity across the included studies, in terms of study design, cost components, and outcome measurements, resulting in limited comparability of results and also making it impossible to carry out a more quantitative synthesis or meta-analysis. Therefore, we adopted a descriptive analytical approach and made targeted comparisons among studies conducted in similar settings or using similar strategies to highlight key patterns and differences. Third, the generalizability of findings may be constrained by the geographic and temporal scope of the included studies. Most were conducted in high-income countries before 2020, which may not reflect evolving health systems or cost structures in LMICs, especially in the post-COVID context. Policymakers should carefully consider the similarities and differences between their own settings and those of the included studies.

Future research should assess the long-term economic impact of COPD screening programs, including not only the optimal frequency and intervals of repeated screening, but also the costs and health outcomes associated with long-term disease management and follow-up care. More real-world implementation studies are needed in LMICs to evaluate the feasibility and affordability of adopting different screening and case-finding strategies. Researchers should also explicitly state the economic perspective adopted (healthcare sector, payer, or societal), and consider including broader societal costs such as patient time and productivity losses whenever feasible, to improve transparency and comparability across studies. As questionnaire screener and portable spirometer were shown promising tools for case detection in the reviewed studies, future research is needed to continue to explore innovative uses of these tools at point of care or in the community settings and evaluate their economic implications. In addition, future economic evaluations could compare standard one-size-fits-all models with risk-stratified approaches powered by machine learning and predictive modeling, and expand COPD screening by integrating the screening for other diseases (e.g., with lung cancer, cardiovascular disease, or asthma) with common risk factors as a comprehensive multi-disease oriented prevention program to guide more efficient and equitable implementation.

In conclusion, this systematic review highlights the potential economic benefits of COPD screening and case-finding, particularly when targeting high-risk populations. Early detection paired with long-term management may reduce healthcare costs and improve outcomes. Policymakers should weigh the immediate costs of screening against the potential long-term savings from reduced disease progression and hospitalization rates. Future efforts should focus on standardizing the reporting of the study design regarding COPD screening programs and expanding research into LMICs and personalized screening to better inform global healthcare policy decisions.

## Supplementary information

Supplementary materials

PRISMA checklist

## Data Availability

All data supporting the ﬁndings of this study are available within the article and its supplementary materials.
