# Supplementary material for: Economic evaluations of screening and case-finding for Chronic Obstructive Pulmonary Disease (COPD): a systematic review
Source: NPJ Prim Care Respir Med. 2026 Jan 21;36:7. doi: 10.1038/s41533-025-00467-1 (PMC12830855; doi:10.1038/s41533-025-00467-1)
Supplement: Supplementary file 1 — Supplementary materials [file 41533_2025_467_MOESM1_ESM.docx]

**SUPPLEMENTARY MATERIALS**

**SUPPLEMENT S1. SEARCH STRATEGY**

**S1.1 Search Strategy**

Studies published up to April 30, 2025 will be retrieved from the following databases: PubMed, EMBASE, Cochrane Library, the National Health Service (NHS) Centre for Reviews and Dissemination (CRD) Database (including the Database of Abstracts of Reviews of Effects (DARE), the NHS Economic Evaluation Database (NHS EED), and the Health Technology Assessment (HTA) Database); with no restrictions on the start time. We used a comprehensive list of search terms for each database; see **Table S1-4** for the detailed search query for each database. No language, study period, or publication restrictions were applied.

**S1.2 Search Terms**

We included three main search term headings:

1. COPD: COPD, airflow obstruction chronic, COAD, chronic airflow obstruction, chronic obstructive lung disease, chronic obstructive pulmonary disease
2. Screening: screening, case finding, detection, spirometry, questionnaire, peak flow
3. Economic evaluation: cost-effectiveness, cost-benefit, cost-utility

Terms under the same main search term headings are combined with OR and are searched in the Title/Abstract/Keywords (if available). MESH or MESH-like terms for the main search term headings were also added. Main search term headings were combined with AND (i.e., COPD AND screening AND Economic evaluation).

**S1.3 Search Query**

**Table S1. The search query for PubMed.**

| **Search number** | **Query** | **Results** |
| --- | --- | --- |
| #1 | "pulmonary disease, chronic obstructive"[MeSH Terms] OR "airflow obstruction chronic"[Title/Abstract] OR "COAD"[Title/Abstract] OR "COPD"[Title/Abstract] OR "chronic airflow obstruction"[Title/Abstract] OR "chronic obstructive lung disease"[Title/Abstract] OR "chronic obstructive pulmonary disease"[Title/Abstract] | 115,927 |
| #2 | "mass screening"[MeSH Terms] OR "screening"[Title/Abstract] OR "screen*"[Title/Abstract] OR "case finding"[Title/Abstract] OR "detection"[Title/Abstract] OR "detect*"[Title/Abstract] OR "spirometry"[Title/Abstract] OR "questionnaire"[Title/Abstract] OR "peak flow"[Title/Abstract] | 4,533,223 |
| #3 | "costs and cost analysis"[MeSH Terms] OR "value of life"[MeSH Terms] OR "cost-effectiveness"[Title/Abstract] OR "cost-benefit"[Title/Abstract] OR "cost-utility"[Title/Abstract] | 333,556 |
| #4 | #1 AND #2 AND #3 | 476 |

**Table S2. The search query for EMBASE.**

| **Search number** | **Query** | **Results** |
| --- | --- | --- |
| #1 | ('chronic obstructive lung disease')/exp OR (('airflow obstruction chronic'):ti,ab,kw) OR ((COAD):ti,ab,kw) OR ((COPD):ti,ab,kw) OR (('chronic airflow obstruction'):ti,ab,kw) OR (('chronic obstructive lung disease'):ti,ab,kw) OR (('chronic obstructive pulmonary disease'):ti,ab,kw) | 232,781 |
| #2 | (screening)/exp OR (('case finding')/exp) OR ((screen*):ti,ab,kw) OR ((detection):ti,ab,kw) OR ((detect*):ti,ab,kw) OR ((spirometry):ti,ab,kw) OR ((questionnaire):ti,ab,kw) OR (('peak flow'):ti,ab,kw) | 6,280,400 |
| #3 | ('economic evaluation')/exp OR ((cost-effectiveness):ti,ab,kw) OR ((cost-benefit):ti,ab,kw) OR ((cost-utility):ti,ab,kw) | 413,525 |
| #4 | #1 AND #2 AND #3 | 754 |

**Table S3. The search query for Cochrane Library.** In Cochrane Review, Clinical Answers, Editorials, and Special Collections.

| **ID** | **Search** | **Hits** |
| --- | --- | --- |
| #1 | MeSH descriptor: [Pulmonary Disease, Chronic Obstructive] explode all trees | 8,032 |
| #2 | (('airflow obstruction chronic'):ti,ab,kw) OR ((COAD):ti,ab,kw) OR ((COPD):ti,ab,kw) OR (('chronic airflow obstruction'):ti,ab,kw) OR (('chronic obstructive lung disease'):ti,ab,kw) OR (('chronic obstructive pulmonary disease'):ti,ab,kw) with Cochrane Library publication date to Jan 2024, in Cochrane Reviews, Clinical Answers, Editorials, Special Collections | 213 |
| #3 | MeSH descriptor: [Mass Screening] explode all trees | 6,033 |
| #4 | ((screen*):ti,ab,kw) OR ((detection):ti,ab,kw) OR ((detect*):ti,ab,kw) OR ((spirometry):ti,ab,kw) OR ((questionnaire):ti,ab,kw) OR (('peak flow'):ti,ab,kw) with Cochrane Library publication date to Jan 2024, in Cochrane Reviews, Clinical Answers, Editorials, Special Collections | 3,033 |
| #5 | MeSH descriptor: [Costs and Cost Analysis] explode all trees | 16,451 |
| #6 | MeSH descriptor: [Value of Life] explode all trees | 50 |
| #7 | ((cost-effectiveness):ti,ab,kw) OR ((cost-benefit):ti,ab,kw) OR ((cost-utility):ti,ab,kw) with Cochrane Library publication date to Jan 2024, in Cochrane Reviews, Clinical Answers, Editorials, Special Collections | 389 |
| #8 | #1 OR #2 in Cochrane Reviews, Clinical Answers, Editorials, Special Collections | 214 |
| #9 | #3 OR #4 in Cochrane Reviews, Clinical Answers, Editorials, Special Collections | 3,033 |
| #10 | #5 OR #6 OR #7 in Cochrane Reviews, Clinical Answers, Editorials, Special Collections | 413 |
| #11 | #8 AND #9 AND #10 in Cochrane Reviews, Clinical Answers, Editorials, Special Collections | 8 |

**Table S4. The search query for the NHS CRD Database**

| **Line** | **Search** | **Hits** |
| --- | --- | --- |
| #1 | MeSH DESCRIPTOR Pulmonary Disease, Chronic Obstructive EXPLODE ALL TREES | 555 |
| #2 | ((airflow obstruction chronic) OR (COAD) OR (COPD) OR (chronic airflow obstruction) OR (chronic obstructive lung disease) OR (chronic obstructive pulmonary disease)) WHERE LPD FROM 01/01/1900 TO 30/04/2025 | 842 |
| #3 | #1 OR #2 | 884 |
| #4 | MeSH DESCRIPTOR Mass Screening EXPLODE ALL TREES | 2,347 |
| #5 | ((screen*) OR (detect*) OR (case finding) OR (spirometry) OR (questionnaire) OR (peak flow)) WHERE LPD FROM 01/01/1900 TO 30/04/2025 | 13,918 |
| #6 | #4 OR #5 | 13,921 |
| #7 | MeSH DESCRIPTOR Costs and Cost Analysis EXPLODE ALL TREES | 17,164 |
| #8 | MeSH DESCRIPTOR Value of Life EXPLODE ALL TREES | 117 |
| #9 | (cost-effectiveness) OR (cost-benefit) OR (cost-utility) WHERE LPD FROM 01/01/1970 TO 30/04/2025 | 19,278 |
| #10 | #7 OR #8 OR #9 | 21,012 |
| #11 | #3 AND #6 AND #10 | 89 |

*Abbreviations*: NHS CRD, the National Health Service Centre for Reviews and Dissemination Database.

**SUPPLEMENT S2. ADDITIONAL DETAILS IN METHODS AND RESULTS**

**S2.1 Standardizing Cost Estimates to 2025 US Dollar Value**

We standardized all reported cost outcomes to 2025 US dollar values to facilitate comparability across studies. International currencies were converted to the US dollar values using the purchasing power parity (PPP) conversion factor from the World Bank,^1^ and all cost estimates were adjusted to the current value in 2025 based on the Consumer Price Index (CPI) in the US.^2^ Parameters used for cost standardization are summarized in the table below.

**Table S5. Parameters used for cost standardization to 2025 US dollars.**

| **Study** | **Reported currency** | **Year** | **Purchasing Power Parity (PPP) conversion factor to year-specific USD** | **Consumer Price Index (CPI) inflation factor to 2025 USD** |
| --- | --- | --- | --- | --- |
| Jones et al., 2005^3^ | UK pound | 2004 | 0.688 | 1.72 |
| Konstantikaki et al., 2011^4^ | Euro | 2008 | 0.708 | 1.50 |
| Thorn et al., 2012^5^ | Swedish Kronor | 2009 | 8.921 | 1.50 |
| Dirven et al., 2013^6^ | Euro | 2013 | 0.798 | 1.38 |
| Haroon et al., 2013^7^ | UK pound | 2011 | 0.706 | 1.44 |
| Jithoo et al., 2013^8^ | Not applicable |  |  |  |
| Tawara et al., 2015^9^ | Japanese yen | 2013 | 101.303 | 1.38 |
| Jordan et al., 2016^10^ | UK pound | 2013 | 0.695 | 1.38 |
| Pan et al., 2021^11^ | UK pound | 2019 | 0.670 | 1.26 |
| Martins et al., 2022^12^ | UK pound | 2019 | 0.670 | 1.26 |
| Mohan et al., 2022^13^ | US dollar | 2019 | 1.000 | 1.26 |
| Lambe et al., 2019^14^ | UK pound | 2015 | 0.692 | 1.36 |
| Du et al., 2021^15^ | Chinese yuan | 2019 | 4.060 | 1.26 |
| Johnson et al., 2021^16^ | Canadian dollar | 2019 | 1.218 | 1.26 |
| Qu et al., 2021^17^ | Chinese yuan | 2018 | 4.094 | 1.28 |
| Mountain et al., 2023^18^ | Canadian dollar | 2021 | 1.163 | 1.21 |
| Chen et al., 2024^19^ | US dollar | 2022 | 1.000 | 1.13 |
| Zhang et al., 2025^20^ | US dollar | 2023 | 1.000 | 1.06 |

Note: Reported costs in international currencies were divided by the Purchasing Power Parity (PPP) conversion factor and then multiplied by the Consumer Price Index (CPI) inflation factor.

**S2.2 Quality Assessment**

We used the Drummond 10-point checklist^21^ to assess the methodological quality of all included studies. Each study was independently evaluated against the ten criteria by one reviewer, with a second reviewer consulted when necessary to resolve uncertainties. **Table S6** summarizes how each study fulfilled the checklist criteria.

**Table S6. Drummond 10-point checklist evaluation for included studies.**

| **Study** | **Research question well defined?** | **Comprehensive description of alternatives?** | **Effectiveness of program established?** | **Important & relevant costs & consequences for each alternative identified?** | **Costs & consequences measured accurately & appropriately?** | **Costs & consequences valued credibly?** | **Costs & consequences adjusted for differential timing?** | **Incremental analysis of costs & consequences performed?** | **Allowance made for uncertainty in estimates?** | **Presentation & discussion of study results include all issues of concern to users?** |
| --- | --- | --- | --- | --- | --- | --- | --- | --- | --- | --- |
| Jones et al., 2005^3^ | Y | P | Y | Y | Y | Y | N | N | N | Y |
| Konstantikaki et al., 2011^4^ | Y | Y | Y | Y | Y | Y | N | Y | N | Y |
| Thorn et al., 2012^5^ | Y | Y | Y | Y | Y | Y | Y | Y | N | Y |
| Dirven et al., 2013^6^ | Y | P | Y | Y | Y | Y | N | N | N | Y |
| Haroon et al., 2013^7^ | Y | Y | Y | Y | Y | Y | Y | Y | Y | Y |
| Jithoo et al., 2013^8^ | Y | Y | Y | Y | Y | Y | N | Y | N | Y |
| Tawara et al., 2015^9^ | Y | P | Y | Y | Y | Y | N | N | N | Y |
| Jordan et al., 2016^10^ | Y | Y | Y | Y | Y | Y | Y | Y | Y | Y |
| Pan et al., 2021^11^ | Y | Y | Y | Y | Y | Y | N | Y | P | Y |
| Martins et al., 2022^12^ | Y | Y | Y | Y | Y | Y | N | Y | N | Y |
| Mohan et al., 2022^13^ | Y | Y | Y | Y | Y | Y | N | Y | P | Y |
| Lambe et al., 2019^14^ | Y | Y | Y | Y | Y | Y | Y | Y | Y | Y |
| Du et al., 2021^15^ | Y | Y | Y | Y | Y | Y | Y | Y | Y | Y |
| Johnson et al., 2021^16^ | Y | Y | Y | Y | Y | Y | Y | Y | Y | Y |
| Qu et al., 2021^17^ | Y | Y | Y | Y | Y | Y | Y | Y | Y | Y |
| Mountain et al., 2023^18^ | Y | Y | Y | Y | Y | Y | N | Y | Y | Y |
| Chen et al., 2024^19^ | Y | Y | Y | Y | Y | Y | Y | Y | Y | Y |
| Zhang et al., 2025^20^ | Y | Y | Y | Y | Y | Y | Y | Y | Y | Y |

*Abbreviations*: Y, yes; N, no; P, partial.

**S2.3 Additional characteristics of included studies**

We summarized additional details on the tools and approaches used for COPD screening and case-finding, the types of economic evaluation conducted, and the key cost and effectiveness components reported in each study. “Questionnaire alone” refers to studies that used questionnaires as the sole screening tool, whereas “questionnaire pre-screening” denotes those that employed questionnaires as an initial step before a portable spirometer. “Portable spirometer alone” refers to studies using handheld spirometers independently, while “portable spirometer following questionnaire” indicates those that applied portable spirometer after an initial questionnaire as a second screening step. Effectiveness was defined, for empirical studies, as the number of detected COPD cases divided by the number of individuals screened, and for modeling studies, as quality-adjusted life-years (QALYs) gained per person.

**Table S7. Additional characteristics of included studies.**

| **Study** | **Screening and case-finding tool and method** | | | | **Cost and effectiveness evaluation** | | |
| --- | --- | --- | --- | --- | --- | --- | --- |
|  | **Questionnaire used** | **Questionnaire alone/pre-screening** | **Portable spirometer used** | **Portable spirometer alone/following questionnaire** | **Economic evaluation perspective** | **Cost components included*** | **Effectiveness^** |
| Jones et al., 2005^3^ |  |  |  |  |  | Program setup, screening delivery | 68/98 (69%) |
| Konstantikaki et al., 2011^4^ | Self-designed questionnaires, COPD-PS | Alone |  |  |  | Program setup, screening delivery | Case-finding: 56 / 201 (27.9%); Open spirometry: 76 / 905 (8.4%) |
| Thorn et al., 2012^5^ |  |  | Mini-spirometer | Alone | Healthcare sector | Screening delivery | 61/305 (20%) |
| Dirven et al., 2013^6^ | RHSQ (CDQ) | Alone |  |  |  | Screening delivery | 9/831 (3%) |
| Haroon et al., 2013^7^ | Self-designed questionnaires | Alone |  |  |  | Program setup, screening delivery | Targeted case-finding: 10/815 (1.2%);  Opportunistic case-finding: 6/819 (0.7%) |
| Jithoo et al., 2013^8^ | Self-designed questionnaires | Alone or pre-screening | Peak flow | Following |  | Screening delivery | CART question only: 7%; A priori question only: 7.8%; A priori question + peak flow: 6.6% |
| Tawara et al., 2015^9^ | 11-Q | Pre-screening | Electronic spirometer | Following |  | Program setup, screening delivery, post screening and management | Initial screening in 2006: 140/4,470 (3.1%); By 2014: 256 diagnosis in total among approx. 8,500 residents |
| Jordan et al., 2016^10^ | Self-designed questionnaires | Alone |  |  | Healthcare sector | Program setup, screening delivery | Active case-ﬁnding: 822/15,378 (5%); Opportunistic case-finding: 370/15,387 (2%) |
| Pan et al., 2021^11^ | CAPTURE, CDQ, C-SBQ, COPD-SQ | Alone and pre-screening | Peak ﬂow, microspirometry | Alone and following |  | Screening delivery | Microspirometry: 216/2,445 (8.8%); Peak flow: 224/2,445 (9.2%); C-SBQ + microspirometry: 166/2,445 (6.8%) |
| Martins et al., 2022^12^ | CAPTURE, CDQ, SBQ, COPD-SQ | Alone and pre-screening | Peak ﬂow, microspirometry | Alone and following |  | Screening delivery | CDQ + peak flow: 20/1,162 (1.7%); CDQ + microspirometry: 23/1,162 (2.0%); SBQ + peak flow: 31/1,162 (2.7%); SBQ + microspirometry:32/1,162 (2.8%); COPD-SQ + microspirometry: 33/1,162 (2.8%); |
| Mohan et al., 2022^13^ | CAPTURE, COLA-6, LFQ | Alone |  |  | Healthcare sector | Screening delivery | Average of three methods in  Nepal: 6.46%; Uganda: 2.6%; Peru: 1.0% |
| Lambe et al., 2019^14^ | Self-designed questionnaires | Alone |  |  | Healthcare sector | Screening delivery, post screening and management | Questionnaire alone, every 3 years: 0.0281 QALYs gained |
| Du et al., 2021^15^ | COPD-PS | Alone |  |  | Payer | Screening delivery, post screening and management | COPD-PS, one-time: 0.28 QALYs gained |
| Johnson et al., 2021^16^ | CDQ | Alone and pre-screening | Hand-held flow meters | Alone and following | Payer | Program setup, screening delivery, post screening and management | Aged ≥ 40 years, CDQ, 3-year intervals: 0.015 QALYs gained |
| Qu et al., 2021^17^ | Not specified | Alone | Portable spirometer | Alone | Payer | Screening delivery, post screening and management | Questionnaire alone: 0.32 QALYs gained; Portable spirometer alone: 0.37 QALYs gained |
| Mountain et al., 2023^18^ | CDQ | Alone and pre-screening | Hand-held flow meters | Alone and following | Payer | Program setup, screening delivery | Effectiveness not appliable |
| Chen et al., 2024^19^ | COPD-SQ | Alone and pre-screening | Portable spirometer | Following | Payer | Program setup, screening delivery, post screening and management | COPD-SQ + portable spirometer, annually: 0.018 QALYs gained |
| Zhang et al., 2025^20^ | COPD-SQ | Pre-screening | Portable spirometer | Following | Healthcare sector | Screening delivery, post screening and management | COPD-SQ + portable spirometer, one-time: 0.021 QALYs gained |

*Cost components include program setup cost, screening delivery cost, Post-screening and management cost, and patient time and productivity cost.

^Effectiveness is described as, for empirical studies, the number of diagnoses from screening divided by the number of eligible individuals; for modeling studies, as QALYs gained per person.

*Abbreviations*: COPD-PS, COPD Population Screener Questionnaire; RHSQ, Respiratory Health Screening Questionnaire; CDQ, COPD Diagnostic Questionnaire; 11-Q, COPD Eleven-Item Pre-Interview Questionnaire; CAPTURE, COPD Assessment in Primary Care To Identify Undiagnosed Respiratory Disease and Exacerbation Risk; C-SBQ, Chinese Symptom-Based Questionnaire; SBQ, Symptom-Based Questionnaire; COPD-SQ, COPD Screening Questionnaire; COLA-6, COPD in Low- and Middle-Income Countries Assessment; LFQ, Lung Function Questionnaire.

**S2.4 Utility, treatment, and exacerbation parameters in modeling studies**

We summarized the parameters for utility, treatment effects, and exacerbation effects used in the included modeling studies. Utility parameters were not applicable to Mountain et al. (2023),^18^ as the study’s primary outcome was budget impact and did not estimate health utility outcomes.

**Table S8. Utility, treatment, and exacerbation parameters in modeling studies.**

| **Study** | **Utility estimation of COPD patients** | **Treatment effect** | **Exacerbation effect** |
| --- | --- | --- | --- |
| Lambe et al., 2019^14^ | Mild COPD: 0.72; Moderate COPD: 0.70; Severe COPD: 0.68; Very severe COPD: 0.59 Source: the Birmingham cohort^22^ | Decrease in mortality rate: OR=0.98; Decrease in exacerbation rate: OR=0.85; Utility gained: 0.0367 | Disutility from severe exacerbation Mild COPD: 0.2398; Moderate COPD: 0.2337; Severe COPD: 0.2265; Very severe COPD: 0.1951 |
| Du et al., 2021^15^ | Mild COPD: 0.81; Moderate COPD: 0.72; Severe/very severe COPD: 0.67; Source: Wilson et al., 2017^23^ | FEV1 improvement: 0.013-0.031L | Mortality of severe exacerbation: 1.28%; Disutility from outpatient exacerbation: 15%; Disutility from inpatient exacerbation: 50% |
| Johnson et al., 2021^16^ | Mild COPD: 0.81; Moderate COPD: 0.72; Severe COPD: 0.68; Very severe: 0.58; Source: Spencer et al.,2005^24^ and Rutten-van Mölken et al., 2009^25^ | Decrease in exacerbation rate: 0.22-0.34; QALY gained: 0.0367 | Disutility from mild-moderate exacerbation: 0.0155-0.0488; Disutility from severe-very severe exacerbation: 0.0655-0.0728 |
| Mountain et al., 2023^18^ | Not appliable | Decrease in exacerbation rate: 0.22-0.34 | Not appliable |
| Qu et al., 2021^17^ | Mild COPD: 0.81; Moderate COPD: 0.72; Severe/very severe COPD: 0.67; Source: Wilson et al., 2017^23^ | FEV1 improvement: 0.013-0.031L | Mortality of severe exacerbation: 1.28%; Disutility from outpatient exacerbation: 15%; Disutility from inpatient exacerbation: 50% |
| Chen et al., 2024^19^ | Mild COPD: 0.81; Moderate COPD: 0.77; Severe COPD: 0.70; Very severe: 0.62; Based on the age-specific baseline utility and adjusted by the COPD GOLD stage Source: Moayeri et al., 2016^26^ and Wu et al., 2016^27^ | Decrease in mortality rate: RR=0.70-0.99; Decrease in exacerbation rate: RR=0.62-0.92; FEV1 improvement: 0.072-0.201L | Disutility from non-severe exacerbation: 14.76%; Disutility from severe exacerbation: 25.04% |
| Zhang et al., 2025^20^ | Mild COPD: 0.88; Moderate COPD: 0.80; Severe COPD: 0.71; Very severe: 0.63; Source: Individual data from National Enjoying Breathing Program | Decrease in mortality rate: RR=0.83-0.86; Decrease in severe exacerbation rate: RR=0.64-67 | Disutility from severe exacerbation Mild COPD: 0.24; Moderate COPD: 0.24; Severe COPD: 0.23; Very severe COPD: 0.20 |

*Abbreviations*: COPD, chronic obstructive pulmonary disease; OR, odds ratio; RR, relative risk; FEV1, forced expiratory volume in 1 second.

**S2.5 Key Study Design Elements**

To improve clarity and comparability, we suggested standardization in the description of key study design elements as outlined in the main text **Table 3**. Accordingly, we summarized the included studies based on these elements (see **Table S9**).

**Table S9. Summary of COPD screening and case-finding study design elements of included studies.**

| **Study** | **Setting** | **Description** | **Study type** | **Risk factor** | | | **Recruitment method** | **Screening** | | **Positive threshold** | **Diagnostic** | | **Frequency** |
| --- | --- | --- | --- | --- | --- | --- | --- | --- | --- | --- | --- | --- | --- |
|  |  |  |  | **Age** | **Smoking** | **Other** |  | **Tool** | **Setting** |  | **Tool** | **Setting** |  |
| Jones et al., 2005^3^ | UK | Structured COPD diagnostic and management | Empirical |  |  |  |  |  | General practices |  | Confirmatory spirometry | General practices | One-time |
| Konstantikaki et al., 2011^4^ | Greece | Open spirometry | Empirical | >30 |  | With chronic respiratory symptoms | Public invitation through local advertisement | Structured questionnaire | Primary care practices | All eligible subjects | Confirmatory spirometry | Primary care practices | One-time |
|  |  | Case-ﬁnding | Empirical | >30 |  | High-risk subjects selected by primary care physicians | During practice visit | Structured questionnaire and screening questionnaire | Primary care practices | All eligible subjects | Confirmatory spirometry | Primary care practices | One-time |
| Thorn et al., 2012^5^ | Sweden | Lung function pre-screening | Empirical | 45–85 | Smoker |  | During primary health care centers (PHCC) visit | Mini-spirometer | PHCC | Various threshold | Confirmatory spirometry | PHCC | One-time |
| Dirven et al., 2013^6^ | Dutch | Population-based early detection | Empirical | 40–70 |  |  | Mail | Screening questionnaire | Mail | Classified as high risk by questionnaire | Confirmatory spirometry | General practice | One-time |
| Haroon et al., 2013^7^ | UK | Targeted case-finding | Empirical | 35-79 | Ever-smokers |  | Mail | Screening questionnaire | Mail | With corresponding respiratory symptoms | Confirmatory spirometry | At the surgery | One-time |
|  |  | Opportunistic case-finding | Empirical | 35-79 | Ever-smokers |  | During GP visit | Screening questionnaire | Mail | With corresponding respiratory symptoms | Confirmatory spirometry | At the surgery | One-time |
| Jithoo et al., 2013^8^ | 14 sites worldwide | Case-finding | Empirical | ≥40 |  |  | Population-based random sample | Screening questionnaire | Face-to-face | Result from prediction model | Confirmatory spirometry |  | One-time |
| Tawara et al., 2015^9^ | Japanese | Systematic intervention | Empirical | 50-89 |  |  | Mail | Screening questionnaire and electronic spirometer | Mail and not mentioned | Recommend threshold | Conﬁrmatory spirometry | Local foundation hospital | 2006 and 2011 |
| Jordan et al., 2016^10^ | UK | Active targeted case-finding | Empirical | 40-79 | Ever-smokers |  | During GP visit and send mail | Screening questionnaire | General practice or by mail | With corresponding respiratory symptoms | Conﬁrmatory spirometry | General practice | One-time |
|  |  | Opportunistic targeted case-finding | Empirical | 40-79 | Ever-smokers |  | During GP visit | Screening questionnaire | General practice | With corresponding respiratory symptoms | Conﬁrmatory spirometry | General practice | One-time |
| Pan et al., 2021^11^ | China | Accuracy of screening test | Empirical | ≥40 |  |  | During community health center visit and through poster or social media | Four questionnaires, peak ﬂow, microspirometry, and their combination | CHC | Recommend threshold | Conﬁrmatory spirometry | Community health centre | One-time |
| Martins et al., 2022^12^ | Brazil | Accuracy of screening test | Empirical | ≥40 |  | Hypertension | During routine consultations at Basic Health Unit (BHU) | Four questionnaires, peak ﬂow, microspirometry, and their combination | BHU | Recommend threshold | Conﬁrmatory spirometry | BHU | One-time |
| Mohan et al., 2022^13^ | Nepal, Peru, Uganda | Screening | Empirical | ≥40 |  |  | Randomly sampled based on census data | Three different screening strategies | Screening visit | Recommend threshold | Conﬁrmatory spirometry | Clinician visit in healthcare system | One-time |
| Lambe et al., 2019^14^ | UK | Systematic case-finding | Modeling | ≥50 | Ever-smokers |  | During GP visit and send mail | Screening questionnaire | General practice or by mail | With corresponding respiratory symptoms | Conﬁrmatory spirometry | General practice | Every 3 years |
| Du et al., 2021^15^ | China | Screening | Modeling | ≥45 | Mainly smoker | Long-term exposure to secondhand smoke/underlying or family history of lung diseases or respiratory symptoms |  | Screening questionnaire | On-site | Recommend threshold | Pulmonary function test | On-site | One-time |
| Johnson et al., 2021^16^ | Canada | Case detection | Modeling | ≥40 | Varied by strategy | Varied by strategy | Primary care | Combination of screening questionnaire and hand-held flow meters | Primary care | Various threshold | Conﬁrmatory spirometry | Primary care | Every 3 or 5 years |
| Qu et al., 2021^17^ | China | Screening | Modeling |  |  | Chronic bronchitis patients |  | Screening questionnaire or portable spirometer |  | Recommend threshold | Conﬁrmatory spirometry |  | One-time |
| Mountain et al., 2023^18^ | Canada | Case detection | Modeling | ≥40 | Varied by strategy | Varied by strategy | Primary care | Combination of screening questionnaire and hand-held flow meters | Primary care | Various threshold | Conﬁrmatory spirometry | Primary care | One-time |
| Chen et al., 2024^19^ | China | Screening | Modeling | ≥35 |  |  |  | Screening questionnaire alone or combined with portable spirometer | On-site | Recommend threshold | Conﬁrmatory spirometry | Qualified hospital | One-time or every 1, 2, 5, 10 years |
| Zhang et al., 2025^20^ | China | Screening | Modeling | ≥40 |  |  | Population-wide recruitment | Screening questionnaire combined with portable spirometer | On-site | Recommend threshold | Conﬁrmatory spirometry | Qualified hospital | One-time |

**REFERENCES**

1. The World Bank. World Bank Open Data. Accessed October 5, 2025. https://data.worldbank.org/

2. U.S. Bureau of Labor Statistics. Consumer Price Index. Accessed October 5, 2025. https://www.bls.gov/cpi/

3. Jones R, Whittaker M, Hanney K, Shackell B. A pilot study of a mobile spirometry service in primary care. *Prim Care Respir J*. 2005;14(3):169-171. doi:10.1016/j.pcrj.2004.12.003

4. Konstantikaki V, Kostikas K, Minas M, et al. Comparison of a network of primary care physicians and an open spirometry programme for COPD diagnosis. *Respir Med*. 2011;105(2):274-281. doi:10.1016/j.rmed.2010.06.020

5. Thorn J, Tilling B, Lisspers K, Jorgensen L, Stenling A, Stratelis G. Improved prediction of COPD in at-risk patients using lung function pre-screening in primary care: a real-life study and cost-effectiveness analysis. *Prim Care Respir J*. 2012;21(2):159-166.

6. Dirven JA, Tange HJ, Muris JW, van Haaren KM, Vink G, van Schayck OC. Early detection of COPD in general practice: implementation, workload and socioeconomic status. A mixed methods observational study. *Prim Care Respir J*. 2013;22(3):338-343. doi:10.4104/pcrj.2013.00071

7. Haroon S, Adab P, Griffin C, Jordan R. Case finding for chronic obstructive pulmonary disease in primary care: a pilot randomised controlled trial. *Br J Gen Pract*. 2013;63(606):e55-e62.

8. Jithoo A, Enright PL, Burney P, et al. Case-finding options for COPD: Results from the burden of obstructive lung disease study. *Eur Respir J*. 2013;41(3):548-555. doi:10.1183/09031936.00132011

9. Tawara Y, Senjyu H, Tanaka K, et al. Value of systematic intervention for chronic obstructive pulmonary disease in a regional Japanese city based on case detection rate and medical cost. *Int J Chron Obstruct Pulmon Dis*. 2015;10:1531-1542. doi:10.2147/copd.S82872

10. Jordan RE, Adab P, Sitch A, et al. Targeted case finding for chronic obstructive pulmonary disease versus routine practice in primary care (TargetCOPD): a cluster-randomised controlled trial. *Lancet Respir Med*. 2016;4(9):720-730. doi:10.1016/S2213-2600(16)30149-7

11. Pan Z, Dickens AP, Chi C, et al. Accuracy and cost-effectiveness of different screening strategies for identifying undiagnosed COPD among primary care patients (=40 years) in China: A cross-sectional screening test accuracy study: Findings from the Breathe Well group. *BMJ Open*. 2021;11(9). doi:10.1136/bmjopen-2021-051811

12. Martins SM, Dickens AP, Salibe-Filho W, et al. Accuracy and economic evaluation of screening tests for undiagnosed COPD among hypertensive individuals in Brazil. *NPJ Prim Care Respir Med*. 2022;32(1). doi:10.1038/s41533-022-00303-w

13. Mohan S, Cárdenas MK, Ricciardi F, et al. Cost-Accuracy Analysis of Chronic Obstructive Pulmonary Disease Screening in Low- and Middle-Income Countries. *Am J Respir Crit Care Med*. 2022;206(3):353-356. doi:10.1164/rccm.202201-0071LE

14. Lambe T, Adab P, Jordan RE, et al. Model-based evaluation of the long-term cost-effectiveness of systematic case-finding for COPD in primary care. *Thorax*. 2019;74(8):730-739. doi:10.1136/thoraxjnl-2018-212148

15. Du M, Hu H, Zhang L, et al. China county based COPD screening and cost-effectiveness analysis. *Ann Palliat Med*. 2021;10(4):4652-4660. doi:10.21037/apm-21-812

16. Johnson KM, Sadatsafavi M, Adibi A, et al. Cost Effectiveness of Case Detection Strategies for the Early Detection of COPD. *Appl Health Econ Health Policy*. 2021;19(2):203-215. doi:10.1007/s40258-020-00616-2

17. Qu S, You X, Liu T, et al. Cost-effectiveness analysis of COPD screening programs in primary care for high-risk patients in China. *NPJ Prim Care Respir Med*. 2021;31(1). doi:10.1038/s41533-021-00233-z

18. Mountain R, Kim D, Johnson KM. Budget impact analysis of adopting primary care-based case detection of chronic obstructive pulmonary disease in the Canadian general population. *CMAJ Open*. 2023;11(6):E1048-e1058. doi:10.9778/cmajo.20230023

19. Chen Q, Fan Y, Huang K, et al. Cost-effectiveness of population-based screening for chronic obstructive pulmonary disease in China: a simulation modeling study. *Lancet Reg Health West Pac*. 2024;46:101065. doi:10.1016/j.lanwpc.2024.101065

20. Zhang T, Ai J, Huang K, et al. Cost-effectiveness of chronic obstructive pulmonary disease population screening in China: based on individual data from WHO Collaborating Centre-initiated ‘Enjoying Breathing Program.’ *BMC Public Health*. 2025;25:1528.

21. Drummond MF, Sculpher MJ, Claxton K, Stoddart GL, Torrance GW. *Methods for the Economic Evaluation of Health Care Programmes*. Oxford university press; 2015. Accessed July 8, 2025. https://books.google.com.sg/books?hl=zh-CN&lr=&id=yzZSCwAAQBAJ&oi=fnd&pg=PP1&dq=Methods+for+the+Economic+Evaluation+of+Health+Care+Programmes&ots=_cRcgI2nGL&sig=TSaidPaKzyRp6Je2Gxx_ZbGc02Q

22. Adab P, Fitzmaurice DA, Dickens AP, et al. Cohort profile: the Birmingham chronic obstructive pulmonary disease (COPD) cohort study. *Int J Epidemiol*. 2017;46(1):23-23.

23. Wilson MR, Patel JG, Coleman A, McDade CL, Stanford RH, Earnshaw SR. Cost-effectiveness analysis of umeclidinium/vilanterol for the management of patients with moderate to very severe COPD using an economic model. *Int J Chron Obstruct Pulmon Dis*. 2017;12:997.

24. Spencer M, Briggs AH, Grossman RF, Rance L. Development of an economic model to assess the cost effectiveness of treatment interventions for chronic obstructive pulmonary disease. *Pharmacoeconomics*. 2005;23(6):619-637. doi:10.2165/00019053-200523060-00008

25. Rutten-van Mölken MPMH, Hoogendoorn M, Lamers LM. Holistic Preferences for 1-Year Health Profiles Describing Fluctuations in Health: The Case of Chronic Obstructive Pulmonary Disease. *PharmacoEconomics*. 2009;27(6):465-477. doi:10.2165/00019053-200927060-00003

26. Moayeri F, Hsueh YSA, Clarke P, Dunt D. Do model-based studies in chronic obstructive pulmonary disease measure correct values of utility? A meta-analysis. *Value Health*. 2016;19(4):363-373.

27. Wu C, Gong Y, Wu J, et al. Chinese Version of the EQ-5D Preference Weights: Applicability in a Chinese General Population. *PLoS One*. 2016;11(10):e0164334. doi:10.1371/journal.pone.0164334
